# The association between electronic cigarette use and respiratory symptoms among university students: A cross-sectional study in Saudi Arabia

**DOI:** 10.18332/tid/203454

**Published:** 2025-05-02

**Authors:** Mawada Q. Alsaedi, Abdulmohsen H. Al-Zalabani

**Affiliations:** 1 Saudi Board of Preventive Medicine, Ministry of Health, Al-Madinah, Saudi Arabia; 2 Department of Family and Community Medicine, Taibah University, Al-Madinah, Saudi Arabia

**Keywords:** young adults, respiratory symptoms, Saudi Arabia, vaping, electronic cigarettes

## Abstract

**INTRODUCTION:**

Electronic cigarette use is on the rise. It is critical to understand the respiratory hazards connected with their use for public health reasons. However, there are limited data concerning this issue in the Middle East and in Saudi Arabia in particular. This study aimed to investigate the association between e-cigarette use and respiratory symptoms among university students in Madinah city, Saudi Arabia.

**METHODS:**

A cross-sectional study was conducted among university students using a self-administered questionnaire with self-reported respiratory symptoms as the primary outcome; a non-probability convenience sampling strategy was used. Emails were sent to all registered students in undergraduate programs between October and November 2024. Appropriate statistical tests were performed on the data, including multivariable logistic regression.

**RESULTS:**

A total of 499 students participated in the study, of which 17.6% were current e-cigarette users and 13.2% were ever users. Respiratory symptoms were most prevalent among current users. Current e-cigarette users and ever users had higher odds of respiratory symptoms than never users (adjusted odds ratio, AOR=2.26; 95% CI: 1.14–4.51 and AOR=2.32; 95% CI: 1.21–4.46, respectively), after adjusting for demographic characteristics, tobacco use, exposure to tobacco and e-cigarettes, and other health factors.

**CONCLUSIONS:**

We found a significant association between e-cigarette use and adverse respiratory symptoms among university students, independent of tobacco use.

## INTRODUCTION

The use of electronic cigarettes has increased rapidly over the years; in 2021, there were an estimated 82 million users globally, including 9.2 million users in the Middle East, raising potential health concerns^1^. Adolescents and young adults tend to use e-cigarettes more often than the general population^2^. Globally, for those aged 12–16 years, the past 30-day prevalence of e-cigarette use is 9.8%^2^.

E-cigarettes were introduced to the market in 2004. Since then, they have become a multi-billion-dollar industry, with North America holding the largest market share in 2021 at an estimated 10.3 billion dollars, while the Middle East market was valued at 490 million dollars^1^. However, the marketing, sales, and content of e-cigarettes remain largely unregulated on a global scale^3^. The Saudi government’s regulations regarding the use and sale of e-cigarettes have changed over the years. In 2015, the importation and sale of these devices were banned^4^. Despite the ban, e-cigarettes were gaining popularity among the community. In 2019, the government repealed the ban and put in place laws governing the sale of electronic cigarettes, which prohibit sales to people aged <18 years^5^. In 2020, e-cigarettes officially entered the Saudi market when the regulations went into effect^6^.

The public widely perceives e-cigarettes as less addictive than conventional cigarettes^7^. Furthermore, the e-cigarette industry presents these products to regulatory and medical communities as replacements for traditional cigarettes that can be used to quit smoking^7^. The 2024 version of the Cochrane living systematic review indicated that using e-cigarettes could be an effective method for quitting smoking with relatively low short-term risks, although the long-term effects are still unknown^8^. However, e-cigarette uptake among non-smokers as well as dual use are concerns, especially since most e-cigarette advertisements are aimed at non-smokers and adolescents^9^. In such cases, e-cigarettes could act as a gateway to tobacco use^9^. Currently, the Saudi Ministry of Health does not officially recommend e-cigarettes as smoking cessation aids^10^.

A recent prospective study found that people who use e-cigarettes report significantly more respiratory symptoms than those who have never vaped, with an odds ratio of 1.8 to 2^11^. The emphasis on the respiratory effects of vaping is justified, given the biological possibility that inhaling flavorings, nicotine, and other compounds may have a negative impact on respiratory health^12^.

Some epidemiological and observational studies among adults and adolescents have reported associations between e-cigarette use and respiratory symptoms such as wheezing, shortness of breath, coughing, and bronchitis symptoms^3,12-23^. A recent systematic review found that such symptoms were prevalent in dual users, smokers who had shifted from traditional cigarettes to e-cigarettes, and those who used e-cigarettes exclusively^9^. However, most of these studies were carried out in developed countries, and there is a lack of comprehensive data pertaining to Saudi Arabia.

There are particular challenges associated with the vaping trend in Saudi Arabia, a nation where tobacco use is highly common, with a prevalence of 19.8%^24^.

First, there is a lack of aggregated data that accurately present the prevalence of e-cigarette use. Second, data regarding the health effects of e-cigarette use on the local community are lacking. The detrimental effects of e-cigarettes seem to be an overlooked aspect of the e-cigarette debate in Saudi Arabia. This knowledge gap hinders the development and application of public health interventions and policies aiming to address health issues related to vaping.

This study aimed to investigate the association between e-cigarette use and respiratory symptoms among university students in the Madinah region of Saudi Arabia.

## METHODS

### Study design, setting, and population

An analytical cross-sectional study was conducted to investigate the association between e-cigarette use and respiratory symptoms among university students in the Madinah region of Saudi Arabia. In one of the major public universities in Madinah, all students who were enrolled in undergraduate programs and were aged >16 years at the time of the study, were invited to participate. The reporting of this study followed the Strengthening the Reporting of Observational Studies in Epidemiology (STROBE) guidelines^25^ (Supplementary file).

### Sampling and sample size

OpenEpi was used to calculate the sample size^26^. Assuming a 30% prevalence of respiratory symptoms among the general population^27^, after applying continuity correction, the study required a total sample size of 426 to achieve a power of 80% for detecting an odds ratio of 1.8^11^ at a two-sided p-value of 0.05. A non-probability convenience sampling strategy was used.

### Data collection

Data were collected between October and November of 2024. A self-administered questionnaire was sent through official channels to all university students who had a registered university email. Two weeks after the initial emails, a reminder was sent to increase the participation rate. Each student received an email invitation to join the study. Those who were interested in participating in the study read the study description and consented to participate by clicking ‘I Agree’ before they proceeded to answer the questions on Microsoft Forms. All items were required to avoid any missing data. The questionnaire took approximately 5–10 minutes to complete. A lottery incentive was provided to promote participation and compensate the participants for their time and effort; 10 participants were randomly chosen to receive gift cards worth 100 Saudi Arabian Riyals each, with this value chosen to avoid coercion.

This study was approved by the Taibah University’s Institutional Review Board (IORG0008716-IRB00010413). Electronic informed consent was obtained from each participant. The participants were informed about the purpose of the study, the voluntary nature of participation, and their ability to opt out at any time. The questionnaire was completed anonymously without any personal identifiers to maintain privacy and confidentiality, as well as to mitigate the potential impact of social desirability bias.

### Questionnaire and study variables

The data were collected using an Arabic self-administered questionnaire that was adapted from previous studies^3,13,20^. Experts in the field of smoking reviewed the content of the questionnaire, and 15 students participated in a pilot and pretest of the questionnaire to assess its comprehensibility. The questionnaire contained four sections that collected information pertaining to demographics, e-cigarette and tobacco use, respiratory symptoms, and health status. The content of each of these sections is described in more detail below.

*Demographics*

This section collected sociodemographic data, including age, sex, college, academic year, and marital status.

*E-cigarette and tobacco use*

Questions about tobacco use were adapted from the Arabic version of the Global Adult Tobacco Survey^28^. E-cigarette use was assessed using the following question: ‘Do you currently use e-cigarettes?’. Those who responded with ‘yes’ were asked, ‘On how many days did you use e-cigarettes in the past 30 days?’. Those who responded with ‘no’ to the first question were asked, ‘Have you used e-cigarettes before even once?’. Those who had used e-cigarettes in the past 30 days were classified as ‘current users’. Those who had used e-cigarettes even once along with those who had used e-cigarettes but not in the past 30 days were classified as ‘ever users’. Those who had never tried e-cigarettes before were classified as ‘never users’, a classification consistent with previous studies^18-20,29^.

Current users were asked about the types of e-cigarettes that they used most frequently (the participants were allowed to select multiple devices) from the following list: pods systems (small, rechargeable devices utilizing pre-filled or refillable cartridges containing e-liquid); vape pens/tanks (refillable, reusable devices with limited modifications, usually featuring a tank holding e-liquid); disposables (such as cigalikes, which are single-use devices pre-filled with e-liquid); mods (devices that provide customization options, enabling users to modify settings and change components); and electronic shishas (or e-shishas, which imitate traditional hookah smoking through electronic heating elements)^22^. Further information was collected about the frequency of e-cigarette use, the age at which use began, the duration of vaping, and the number of puffs per day.

Furthermore, the self-reported data were used to assess the pack-equivalent years of e-cigarette use, which is a measure similar to the cigarette pack-years metric that Chaiton et al.^23^ proposed. It was calculated from: 1) The number of puffs per day (i.e. ‘When you vape, how many puffs do you take?’) divided by 10 (the standard number of puffs in a traditional cigarette); 2) The participants were asked how many days they had used e-cigarettes in the past 30 days, and the average daily use was calculated by dividing that number by 30; and 3) In order to calculate the number of years that the participants had been using e-cigarettes, they were asked, ‘How old were you when you first tried vaping?’, and the response was subtracted from their current age. The formula for pack-equivalent years of e-cigarette use^23^ was:

Pack-equivalent years = (Puffs per day/10) × (Days vaped per month/30) × (Years of vaping)/20

where we divided by 20 to convert to packs (20 cigarettes per pack).

*Respiratory symptoms*

This section of the questionnaire assessed respiratory symptoms in the past 12 months using ‘yes/no’ questions. Based on prior research on respiratory symptoms among e-cigarette users, the study measured four different respiratory symptoms^3,13,16-18,20,22^: wheezing (‘Have you at any time during the last 12 months had wheezing or whistling in your chest?’), shortness of breath (‘Are you troubled by shortness of breath when hurrying on level ground or walking up a slight hill?’), chronic cough (‘Have you had a long-standing cough during the last year?’), and bronchitis symptoms (‘Do you bring up phlegm on most days for periods of at least three months?’ and ‘Have you had such periods during at least two successive years?’).

An affirmative response to any of these questions indicated the presence of respiratory symptoms.

*Health status*

This section contained questions about the health status of the participants and other covariates which were chosen based on previously published research, including self-reported diagnoses of chronic obstructive pulmonary disease (COPD), hay fever, asthma, and COVID-19^12,22^. The participants were asked to report whether they had exercised in the past 30 days^18^ and whether they had secondhand exposure to tobacco and e-cigarettes^30^. They were also asked to fill in their weight (kg) and height (cm), which enabled the calculation of the participants’ body mass index (BMI, kg/m^2^)^16^.

### Statistical analysis

Statistical analysis was conducted using IBM SPSS Statistics (Version 27), and figures were generated using Microsoft Excel. The normality of the quantitative variables was assessed using Shapiro– Wilk and Kolmogorov–Smirnov tests. All of the quantitative variables in the study were skewed, so medians and interquartile ranges (IQRs) were used to summarize the data, while frequencies and proportions were used to summarize the qualitative data. The chi-squared test was conducted to compare the prevalence of adverse respiratory symptoms among never, ever, and current e-cigarette users. Spearman correlation was used to assess the relationship between the pack-equivalent years and the number of respiratory symptoms. Multivariable logistic regression was used to analyze the relationship between e-cigarette use (independent variable) and any respiratory symptoms (dependent variable). Demographic characteristics, tobacco use, secondhand exposure to e-cigarettes, secondhand exposure to tobacco, asthma, COVID-19, hay fever, exercise, and BMI were adjusted for and included in the model based on previously published research, with the results expressed as adjusted odds ratios (AORs) with 95% confidence intervals (CIs).

Given the strong association between self-reported respiratory symptoms and gender, we conducted a further analysis to evaluate whether gender modifies the association between e-cigarette use and respiratory symptoms. To assess this potential effect modification, we conducted a logistic regression model, including multiplicative interaction terms between the exposure (e-cigarette use status) and gender. Effect modification was considered present if the p-value for the interaction term was <0.05. To further explore the relationship within each gender stratum, two separate adjusted models were performed for males and females. The significance level for all the analyses was set at a p<0.05.

## RESULTS

### Sample characteristics

A total of 499 students with a median age of 21 years (IQR: 19–22) participated in the study. Most of the participants were female (267; 53.5%), single (481; 96.4%), of normal weight (246; 49.3%), and in their second academic year (147; 29.5%). In total, 45 (9.0%) students self-reported an asthma diagnosis, 170 (34.1%) self-reported a history of COVID-19, and none of the participants self-reported a diagnosis of COPD. Students who reported tobacco use in the previous 30 days accounted for 11.4% of the total sample. In total, 165 students (33.1%) reported secondhand exposure to tobacco at least once a week, while 75 (15.0%) reported secondhand exposure to e-cigarette aerosol. The characteristics of the study participants are summarized in Table 1.

**Table 1:** Sociodemographic characteristics and health status of participants according to e-cigarette use status, a cross-sectional study, Al-Madinah, Saudi Arabia, 2024 (N=499)

*Characteristics*	*Total* *(N=499)* *n (%)*	*Never users* *(N=345)* *n (%)*	*Ever users* *(N=66)* *n (%)*	*Current users* *(N=88)* *n (%)*	p*
**Age** (years)					**0.013**
16–18	56 (11.2)	45 (13.0)	2 (3.0)	9 (10.2)	
19–21	275 (55.1)	193 (55.9)	40 (60.6)	42 (47.7)	
22–24	136 (27.3)	91 (26.4)	15 (22.7)	30 (34.1)	
≥25	32 (6.4)	16 (4.6)	9 (13.6)	7 (8.0)	
**Gender**					**<0.001**
Male	232 (46.5)	132 (38.3)	44 (66.7)	56 (63.6)	
Female	267 (53.5)	213 (61.7)	22 (33.3)	32 (36.4)	
**Academic year**					**0.533**
1	87 (17.4)	63 (18.3)	12 (18.2)	12 (13.6)	
2	147 (29.5)	108 (31.3)	15 (22.7)	24 (27.3)	
3	94 (18.8)	61 (17.7)	15 (22.7)	18 (20.5)	
4	96 (19.2)	62 (18.0)	17 (25.8)	17 (19.3)	
>4	75 (15.0)	51 (14.8)	7 (10.6)	17 (19.3)	
**Marital status**					**0.113**
Single	481 (96.4)	333 (96.5)	61 (92.4)	87 (98.9)	
Married	18 (3.6)	12 (3.5)	5 (7.6)	1 (1.1)	
**Past 30-days tobacco use**	57 (11.4)	0 (0)	13 (19.7)	44 (50.0)	**<0.001**
**Secondhand exposure to tobacco**	165 (33.1)	94 (27.2)	30 (45.5)	41 (46.6)	**<0.001**
**Secondhand exposure to e-cigarettes**	75 (15.0)	39 (11.3)	11 (16.7)	25 (28.4)	**<0.001**
**BMI** (kg/m^2^)					**0.341**
Underweight (<18.5)	100 (20.0)	74 (21.4)	9 (13.6)	17 (19.3)	
Normal weight (18.5–24.9)	246 (49.3)	172 (49.9)	31 (47.0)	43 (48.9)	
Overweight (25–29.9)	77 (15.4)	45 (13.0)	15 (22.7)	17 (19.3)	
Obese (>30)	76 (15.2)	54 (15.7)	11 (16.7)	11 (12.5)	
**Previous health conditions**					
Asthma	45 (9.0)	28 (8.1)	6 (9.1)	11 (12.5)	**0.440**
COVID-19	170 (34.1)	120 (34.8)	23 (34.8)	27 (30.7)	**0.761**
Hay fever (allergic rhinitis)	100 (20.0)	71 (20.6)	12 (18.2)	17 (19.3)	**0.890**
Exercise, yes	360 (72.1)	243 (70.4)	50 (75.8)	67 (76.1)	**0.443**

* Calculated by chi-squared test or Fisher’s exact test. BMI: body mass index.

### Electronic cigarette use patterns

Overall, 88 (17.6%) students were current e-cigarette users, of whom 44 (50%) had used tobacco in the previous 30 days. The median age of initiating e-cigarette use was 18 years (IQR: 17–19), with more than half of the participants reporting a duration of e-cigarette use of >2 years (48; 54.5%). The most used e-cigarette devices were pod systems (56.8%), followed by disposable e-cigarettes (44%), and then vape pens/tanks (23.9%) (Table 2).

**Table 2:** E-cigarette use patterns among current users, a cross-sectional study, Al-Madinah, Saudi Arabia, 2024 (N=88)

*Variable*	*Median (IQR)*
**Vaping status**	
Age started vaping (years)	18 (17–19)
Number of puffs per day	50 (20–250)
Number of days vaped in the past 30 days	30 (22–30)
Pack-equivalent years^a^	0.51 (0.20–3.75)
***Variable***	***n (%)***
**Most frequent e-cigarette device among current users^b^**	
Pods system	50 (56.8)
Vape pens/tank	21 (23.9)
Disposables	39 (44.3)
Mods	1 (1.1)
Electronic shisha	16 (18.2)
**Duration of vaping**	
<1 month	4 (4.5)
1–3 months	3 (3.4)
4–11 months	9 (10.2)
1–2 years	24 (27.3)
>2 years	48 (54.5)
**Past 30-days tobacco use**	44 (50.0)

a Pack-equivalent years were calculated as: (Puffs per day/10)×(Days vaped per month/30)×(Years of vaping)/20.

b Participants were allowed to select multiple devices. IQR: interquartile range.

### Prevalence of respiratory symptoms

**Figure 1:**
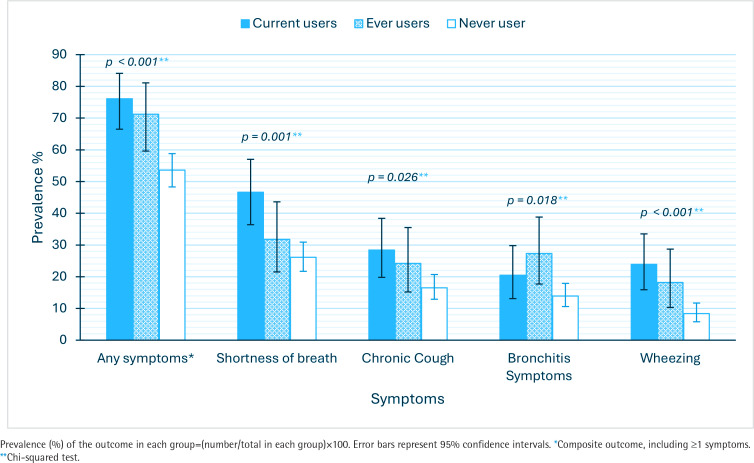
Prevalence of self-reported respiratory symptoms according to e-cigarettes use, a cross-sectional study, Al-Madinah, Saudi Arabia, 2024 (N=499)

Figure 1 depicts the prevalence of self-reported respiratory symptoms according to e-cigarette use status. Overall, respiratory symptoms were more prevalent among current e-cigarette users, except for bronchitis symptoms, which were higher among ever users, with a prevalence of 27.3% versus 20.5% for current users and 13.9% for never users. Wheezing [χ^2^ (2)=17.714, p<0.001], shortness of breath [χ^2^ (2)=13.224, p=0.001], chronic cough [χ^2^ (2)=7.299, p=0.026], bronchitis symptoms [χ^2^ (2)=8.064, p=0.018], and any respiratory symptoms [χ^2^ (2)=18.836, p<0.001] showed statistically significant associations with e-cigarette use. Additionally, there was a significant positive weak correlation between the number of reported symptoms and the pack-equivalent years measure (ρ=0.191, p<0.001).

### Associations between reported respiratory symptoms and e-cigarette use

In the unadjusted analyses, the odds of experiencing any respiratory symptoms were higher among current users (OR=2.76; 95% CI: 1.62–4.71) and ever users (OR=2.14; 95% CI: 1.21–3.79) compared to never users. Furthermore, significant associations were observed between experiencing any respiratory symptoms and secondhand exposure to tobacco, secondhand exposure to e-cigarettes, tobacco use, female sex, and the 22–24 years age group. The complete univariate analysis results are presented in Table 3.

**Table 3:** Unadjusted odds ratio for respiratory symptoms by participants’ characteristics, a cross-sectional study, Al-Madinah, Saudi Arabia, 2024 (N=499)

*Variable*	*Any respiratory symptoms ^a^*		*OR (95% CI)*	*p*
	*n*	*%^b^*		
**E-cigarette use status**				
Never ®	185	53.6	1	
Ever	47	71.2	2.14 (1.21–3.79)	**0.009**
Current	67	76.1	2.76 (1.62–4.71)	**<0.001**
**Past 30-days tobacco use**	48	84.2	4.06 (1.94–8.48)	**<0.001**
**Secondhand exposure to tobacco**	112	67.9	1.66 (1.12–2.46)	**0.011**
**Secondhand exposure to e-cigarettes**	57	76	2.38 (1.36–4.19)	**0.003**
**Age** (years)				
16–18 ®	27	48.2	1	
19–21	160	58.2	1.49 (0.84–2.66)	**0.172**
22–24	90	66.2	2.10 (1.11–3.96)	**0.022**
≥25	22	68.8	2.36 (0.95–5.89)	**0.065**
**Gender**				
Male ®	120	51.7	1	
Female	179	67	1.89 (1.32–2.73)	**<0.001**
**Marital status**				
Single ®	285	59.3	1	
Married	14	77.8	2.41 (0.78–7.42)	**0.126**
**Previous health conditions**				
Asthma	34	75.6	2.20 (1.08–4.46)	**0.028**
COVID-19	112	65.9	1.47 (0.99–2.16)	**0.051**
Hay fever	71	71.0	1.84 (1.14–2.95)	**0.012**
Exercise, yes	208	57.8	0.72 (0.48–1.09)	**0.117**
**BMI**				
Underweight ®	58	58	1	
Normal weight	139	56.5	0.94 (0.59–1.51)	**0.799**
Overweight	50	64.9	1.34 (0.73–2.48)	**0.349**
Obese	52	68.4	1.57 (0.84–2.93)	**0.158**

a Composite outcome, including ≥1 symptoms (wheezing, chronic cough, shortness of breath, bronchitis symptoms).

b Prevalence (%) of the outcome in each group=(number/total in each group)×100. BMI: body mass index. ® Reference categories.

The adjusted analysis showed that after controlling for other variables in the model, e-cigarette use was significantly associated with respiratory symptoms, with both current users (AOR=2.26; 95% CI: 1.14–4.51) and ever users (AOR=2.32; 95% CI: 1.21–4.46) showing higher odds of respiratory symptoms than never users (Table 4). Furthermore, a sensitivity analysis that excluded students with a history of COVID-19 and asthma, showed similar results in terms of the association between e-cigarette use and respiratory symptoms (Table 5).

**Table 4:** Adjusted odds ratios for respiratory symptoms by participant characteristics, a cross-sectional study, Al-Madinah, Saudi Arabia, 2024 (N=499)

*Variable*	*AOR (95% CI)*	*p*
**E-cigarette use status**		
Never ®	1	
Ever	2.32 (1.21–4.46)	**0.012**
Current	2.26 (1.14–4.51)	**0.020**
**Past 30-days tobacco use**	3.79 (1.50–9.62)	**0.005**
**Secondhand exposure to tobacco**	1.34 (0.83–2.16)	**0.224**
**Secondhand exposure to e-cigarettes**	1.39 (0.72–2.72)	**0.325**
**Age** (years)		
16–18 ®	1	
19–21	1.49 (0.79–2.83)	**0.214**
22–24	2.17 (1.07–4.37)	**0.031**
≥25	1.24 (0.39–3.92)	**0.718**
**Gender**		
Male ®	1	
Female	3.46 (2.21–5.39)	**<0.001**
**Marital status**		
Single ®	1	
Married	2.34 (0.55–9.95)	**0.248**
**Previous health conditions**		
Asthma	2.94 (1.31–6.56)	**0.009**
COVID-19	1.69 (1.09–2.59)	**0.017**
Hay fever	2.13 (1.25–3.62)	**0.005**
Exercise, yes	0.59 (0.37–0.95)	**0.030**
**BMI**		
Underweight ®	1	
Normal weight	1.05 (0.62–1.78)	**0.868**
Overweight	1.41 (0.70–2.83)	**0.336**
Obese	2.29 (1.11–4.70)	**0.024**

AOR: adjusted odds ratio; adjusted for e-cigarettes use, age, gender, marital status, secondhand exposure to tobacco, secondhand exposure to e-cigarettes, tobacco use, asthma, COVID-19, hay fever, exercise, and BMI. ® Reference categories. BMI: body mass index.

**Table 5:** Sensitivity analysis excluding students with asthma and COVID-19, a cross-sectional study, Al-Madinah, Saudi Arabia, 2024 (N=294)

*Variable*	*AOR (95% CI)*	*p*
**E-cigarette use status**		
Never ®	1	
Ever	2.51 (1.11–5.71)	**0.028**
Current	2.89 (1.16–7.18)	**0.022**
**Past 30-days tobacco use**	2.86 (0.93–8.83)	**0.068**
**Secondhand exposure to tobacco**	1.08 (0.59–1.96)	**0.800**
**Secondhand exposure to e-cigarettes**	1.85 (0.76–4.50)	**0.176**
**Age** (years)		
16–18 ®	1	
19–21	1.24 (0.52–2.96)	**0.627**
22–24	1.99 (0.78–5.11)	**0.153**
≥25	1.70 (0.36–7.99)	**0.500**
**Gender**		
Male ®	1	
Female	3.03 (1.72–5.34)	**<0.001**
**Marital status**		
Single ®	1	
Married	2.68 (0.41–17.65)	**0.305**
**Hay fever**	2.43 (1.19–4.97)	**0.015**
**Exercise, yes**	0.66 (0.37–1.17)	**0.151**
**BMI**		
Underweight ®	1	
Normal weight	0.85 (0.44–1.64)	**0.632**
Overweight	1.16 (0.47–2.88)	**0.748**
Obese	1.37 (0.57–3.29)	**0.488**

AOR: adjusted odds ratio; adjusted for e-cigarettes use, age, gender, marital status, secondhand exposure to tobacco, secondhand exposure to e-cigarettes, tobacco use, hay fever, exercise, BMI. ® Reference categories. BMI: body mass index.

In our assessment of gender as a potential effect modifier, the overall interaction term was significant (p=0.024 for interaction). In the stratified analysis, male students who reported ever and current e-cigarettes use had higher odds of any respiratory symptoms compared with never users (AOR=5.09; 95% CI: 2.06–12.57 and AOR=3.98; 95% CI: 1.60–9.88, respectively), after controlling for other variables in the model (Supplementary file Table 1). While there was no significant statistical association noted in females (Supplementary file Table 1).

## DISCUSSION

In this study, we aimed to investigate the association between e-cigarette use and respiratory symptoms among Saudi university students in the Madinah region. Overall, respiratory symptoms were most prevalent among current e-cigarette users, followed by ever users, and were least prevalent among never users. Current and ever e-cigarette users were about 2.3 times more likely to report any respiratory symptoms compared to never users. These odds persisted even after we controlled for other covariates, including tobacco use. This finding is consistent with other studies that found associations between e-cigarette use and respiratory symptoms among adults^3,9,11,12,14,18,19,22,23^ and adolescents^11,15,20-23^. Some previous studies have examined the relationship between e-cigarette use and respiratory outcomes from the perspective of diagnosed conditions such as asthma^21^ and COPD^31^. In this study, the focus was on respiratory symptoms rather than diagnosed diseases, as a broader range of effects can be captured by looking at symptoms, including those that might not yet show up as recognized conditions. Additionally, as expected, there was no self-reported COPD diagnosis in this study sample of young adults, as COPD is predominantly diagnosed among those aged >40 years^31^.

Our findings align with mechanistic evidence from experimental studies. Both animal models and laboratory research have demonstrated that e-cigarettes can affect respiratory function through multiple pathways, including altered gene expression, impaired ciliary function in nasal and bronchial tissue, and enhanced inflammatory responses^32^. The mechanisms by which e-cigarettes cause these changes are not yet well understood, but they are likely related to the composition of the ingredients used in e-cigarette products and the harmful inhaled substances, such as carbonyls, volatile organic compounds, nicotine, trace metals, microbial toxins, and flavorings^33^.

Regulations that recently went into effect in Saudi Arabia restrict the sale of e-cigarette products to individuals younger than 18 years and mandate that e-cigarette products receive authorization from the Saudi Food and Drug Administration (SFDA) before entering the market^5^. However, there is currently no obligation for e-cigarette manufacturers to provide an ingredient list on the product label. As a result of this policy gap, e-cigarette users could be denied essential information about the contents of these products, especially considering how rapidly these products are changing and how many new varieties are becoming available in the market.

Our secondary effect modification analysis suggests that the association between e-cigarette use and any respiratory symptoms was modified by gender. We found that the effect of e-cigarette use on respiratory symptoms was larger and statistically significant in male students in contrast to female students, which could be due to differences in vaping patterns, biological responses, cultural norms, or behavioral factors between males and females. This finding, however, should be interpreted with caution due to the exploratory nature of the analysis. Further larger studies with robust analysis are needed to better understand gender specific effects of e-cigarette use and their potential health implication.

In our study, the most used e-cigarette devices among current users were pod systems. Given the small number of current users in our sample, we were unable to conduct further subgroup analyses based on device type. A cross-sectional analysis of four studies from the United States showed a significant association between e-cigarette use and shortness of breath and bronchitis symptoms, and the type of e-cigarette device did not significantly influence the strength of the associations^22^. In contrast, a retrospective study among Canadian youth found that among current vapers, those using pod systems were more likely to experience respiratory symptoms after adjusting for dose^23^.

In this study, 30.9% of the respondents reported e-cigarette use; 17.6% were current users and 13.2% were ever users. Similarly, a study in Saudi Arabia by Althobaiti et al.^[Bibr CIT0034]34^ reported an e-cigarette use prevalence of 26%. Another study conducted in Saudi Arabia by Alhumaidan et al.^35^ reported a lower rate of 10.5%. In contrast, Saudi Arabia’s latest tobacco survey reported that just 2.2% of adults used e-cigarettes^24^. The varying prevalence could be explained by the different age groups; our study focused on university students who were in their early twenties, while the national tobacco survey focused on adults with a median age of 36.7 years^24^. Additionally, the national data may underestimate the true prevalence because the last national survey was carried out in May 2019, and e-cigarettes did not legally enter the Saudi market until early 2020. The higher prevalence of e-cigarette use among young adults may be attributed to several factors. Marketing strategies frequently target adolescents and young adults, often portraying e-cigarette use as socially desirable and fashionable. Additionally, e-cigarettes are easy to purchase in retail stores and on online platforms, potentially increasing their adoption among the youth. Furthermore, vaping is not as socially disapproved of as smoking conventional cigarettes is.

The current legal framework for e-cigarettes in Saudi Arabia falls under different bodies, such as the anti-smoking law; the Saudi Standards, Metrology and Quality Organization; and the SFDA^5,6^. These regulatory bodies oversee the technical and regulatory aspects of e-cigarettes, including public use restrictions, sales, importation, and certain product standards (such as nicotine content limits and ingredient bans). However, there are no laws controlling sponsorship, promotion, or advertising for e-cigarettes, which may allow unrestricted marketing. Since there are no clear regulations on online sales, enforcing age restrictions might be challenging. Additionally, health warnings are only required to be presented in text, which may be less effective than pictorial warnings. These policy gaps, especially in terms of online sales and advertising, have the potential to increase the accessibility and appeal of e-cigarettes, especially among non-smokers and young people, which would undermine public health goals.

### Limitations

Even though this study provides insights into the associations between e-cigarette use and respiratory symptoms among Saudi youths, it has its limitations. First, the inherent limitation of the cross-sectional study design is that temporality cannot be established. Second, the study depends on self-reported data, which are prone to recall bias. Further studies using objective measures are needed to better assess the health impact of e-cigarettes. In addition, given the non-probability sample, the generalizability of the study findings is limited.

Baseline risk should be considered when interpreting our study findings, as those with respiratory issues might feel more inclined to use e-cigarettes than other methods of smoking (reverse causality). However, in the sensitivity analyses, which excluded participants with a prior history of asthma and COVID-19, the association between e-cigarette use and respiratory symptoms did not change, which could make this explanation less likely. Even though we adjusted for current tobacco use, residual confounding caused by prior tobacco use cannot be ruled out.

## CONCLUSIONS

We found a significant association between e-cigarette use and adverse respiratory symptoms among our cross-sectional sample of university students, independent of tobacco use. Further longitudinal large-scale studies on exclusive e-cigarette users should be conducted to assess temporality, preferably in the local community of Saudi Arabia.

## Supplementary Material



## Data Availability

The data supporting this research are available from the authors on reasonable request.
